# Study on the Effect of Deposited Graphene Oxide on the Fatigue Life of Austenitic Steel 1.4541 in Different Temperature Ranges

**DOI:** 10.3390/ma15010065

**Published:** 2021-12-22

**Authors:** Barbara Nasiłowska, Zdzisław Bogdanowicz, Paweł Bogusz, Aneta Bombalska, Zygmunt Mierczyk

**Affiliations:** 1 Institute of Optoelectronics, Military University of Technology, gen. S. Kaliskiego 2, 00-908 Warsaw, Poland; aneta.bombalska@wat.edu.pl (A.B.); zygmunt.mierczyk@wat.edu.pl (Z.M.); 2 Faculty of Mechanical Engineering, Military University of Technology, gen. S. Kaliskiego 2, 00-908 Warsaw, Poland; zdzislaw.bogdanowicz@wat.edu.pl (Z.B.); pawel.bogusz@wat.edu.pl (P.B.)

**Keywords:** graphene oxide, austenitic steel, steel 1.4541, fatigue life

## Abstract

This paper presents the effect of deposited graphene oxide coating on fatigue life of austenitic steel 1.4541 at 20 °C, 100 °C, and 200 °C. The study showed a decrease in the fatigue life of samples with a deposited graphene oxide layer in comparison with reference samples at 20 °C and 100 °C. However, an increase in fatigue life of samples with a deposited graphene oxide layer in comparison with reference samples occurred at 200 °C. This relationship was observed for the nominal stress amplitude of 370 and 420 MPa. Measurements of temperature during the tensile failure of the sample and microfractographic analysis of fatigue fractures were performed. Tests have shown that graphene oxide deposited on the steel surface provides an insulating layer. A higher temperature of the samples with a deposited graphene oxide layer was observed during fracture compared to the reference samples.

## 1. Introduction

Improvements in mechanical properties after the addition of graphene or its derivatives to composites have resulted from many research projects [1,2,3,4]. The results of fatigue life studies presented in work by Demir et al. [2], Rafiee et al. [3], and Li et al. [4], among others, have shown that a small addition of graphene or its derivatives in the volume of a composite results in an improvement in its mechanical properties.

Li et al. [4] showed that the addition of %wt. 0.075 multilayer graphene into concrete fills the voids in the porous structure, leading to a 49.3% increase in fatigue life. Moreover, the content of ~0.2% graphene in a composite consisting of epoxy fibers increases the flexural fatigue life by up to 1200 times [3]. Yavari et al. [5] showed that addition of graphene caused a ~3–5-fold increase in the fatigue life of fiberglass/epoxy composites.

Najfi et al. [6] conducted an analysis of the effect of graphene oxide functional groups on fatigue strength. They indicated that graphene oxide with a low degree of functionalization (<15%) exhibited a significantly higher fatigue life compared to graphene. The addition of ether groups resulted in delayed crack growth and increased fatigue strength [6].

Taking into account the increase in fatigue life of composites containing graphene [2,3,4,5,6,7] and its structural properties, including a high fracture strength of 125 GPa [7], the effect of a deposited layer of graphene and its derivatives on the mechanical properties of structural materials is an interesting research issue. Studies presented in [1] have shown that deposition of graphene oxide on the surface of St3S steel can contribute to an increase in corrosion resistance.

Currently, the most well-known method for depositing graphene on the surface of (mainly) copper is chemical vapor deposition (CVD) [8,9,10]. However, in many research centers, there are attempts to deposit graphene and its derivatives on the surface of construction materials using other methods, such as those with the use of an aerosol [11]. Studies performed on 304 steel bolts showed that hybrid graphenation (shot peening of a graphene layer deposited on the steel surface) leads to an increase in fatigue life of 42–275% (depending on the stress amplitude level) [12].

However, to date, fatigue life tests at elevated temperatures (20 °C, 100 °C, and 200 °C) of flat specimens made of 1.4541 steel with a deposited graphene oxide (GO) layer have not been performed. Therefore, taking into account the operation of many machine parts and structural elements at elevated temperatures, the aim of this work was to determine the effect of a deposited graphene oxide layer on mechanical properties.

## 2. Materials and Methods

Static tension tests and fatigue life tests were performed on flat paddle specimens of 5 mm thickness made of 1.4541 steel according to the following designations: BM—base material (as supplied) (Section 2.1), and BM + GO—base material with a deposited graphene oxide layer (Section 2.1, Section 2.2 and Section 2.3).

### 2.1. Steel 1.4541

**Figure 1:**
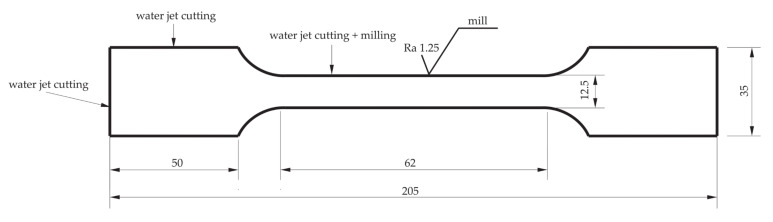
Dimensions (mm) of BM (Base Material) and BM + GO (Base Material + Graphene Oxide) samples subjected to mechanical and fatigue testing.

The dumbbell specimens (Figure 1) were made of 1.4541 steel (according to the designations EN 1.4541, PN 1H18N10T, AISI 321, X5CrNi18-10, SS 2337), whose percentage content of alloying elements was as follows: Ni 9.0–12.0; Cr 17.0–19.0; Mn < 2.0; C < 0.08; Si < 1.0; *p* < 0.045; S < 0.015; T < 0.70; and Fe = bal. Test specimens were cut by the Water jet method from a 2000 × 1000 × 5 mm^3^ metal sheet perpendicular to the rolling direction. The inner edges of the specimens were subjected to a milling process as shown in Figure 1.

### 2.2. Graphene Oxide (GO)

Graphene oxide (GO) flakes dispersed in water were obtained from flake graphite by a modified Hummers method (Department of Chemical Synthesis and Flake Graphene, Łukasiewicz Research Network—Institute of Electronic Materials Technology, Warsaw, Poland). The concentration of graphene oxide water dispersion was 10 g/L. Flake size was 3–10 µm. The detailed synthesis method was described previously in [11,12,13].

### 2.3. Methodology of Deposition of the Graphene Oxide on the Surface

**Figure 2:**
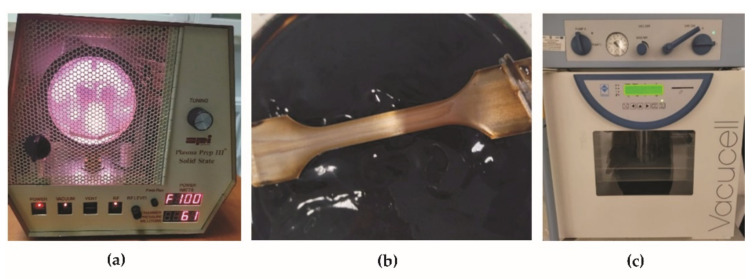
Deposition process of graphene oxide on BM + GO samples; activation and plasma purification (**a**), immersion in dispersed graphene oxide aqueous suspension (**b**), vacuum drying (**c**).

In the first step of graphene oxide deposition on BM + GO samples, surface activation and cleaning with RF (radio frequency) plasma was performed using a Plasma Prep III device (Garfield Ave, West Chester, PA, USA) (Figure 2a). The following parameters were used: 100 W for 30 min. The interaction of the plasma with the steel surface causes an increase in hydrophilicity [14,15] so that the aqueous suspension better wets the steel surface promoting the dissolution of graphene oxide flakes on the surface. This process, in addition to cleaning the surface, is aimed at the adhesive incorporation of graphene oxide particles into the surface layer of steel. Immediately after removal from the plasma chamber, the samples were placed in a Petri dish with a dispersed graphene oxide aqueous suspension for 10 min (Figure 2b). In the last step, the excess suspension was removed mechanically and the samples were placed in a vacuum dryer Vacucell 22 L (BMT Medical Technology s.r.o., Brno-Zábrdovice, Czech Republic) at 40 °C for 24 h (Figure 2c).

### 2.4. Static and Fatigue Tensile Tests

**Figure 3:**
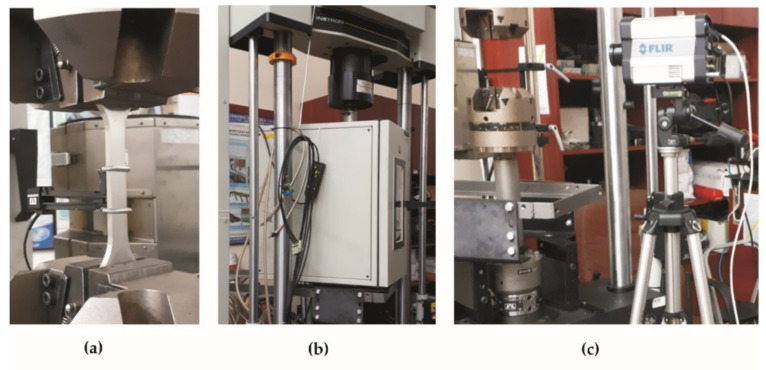
Instron 8862 test stand (**a**), climate chamber (**b**) FLIR SC6000 camera (**c**).

Static tensile and fatigue tests were performed using an Instron 8862 (Norwood, MA, USA) electromechanically driven testing machine with a force range of ±100 kN (Figure 3a) coupled to a climate chamber (Figure 3b). An Instron model 2620-604 extensometer (Norwood, MA, USA) was used to measure strain, with a 25 mm measurement base. The tests were performed at temperature levels of 20, 100, and 200 °C for BM and BM + GO samples.

Table 1 shows the values of the maximum stress (in MPa) and the maximum, minimum, mean, and amplitude of the stress cycle applied to the test specimens (in kN).

Fatigue tests were carried out for different stress levels Rm = 0.6, 0.7, 0.8, and 0.9 at maximum stress levels of 370, 420, 500, and 540 MPa respectively (Table 1). The cycle asymmetry factor was R = 0.1, while the load frequency was 1 Hz.

During static and fatigue measurements at 20 °C, the temperature distribution was performed using a FLIR (Teledyne FLIR, Wilsonville, OR, USA) model SC6000 thermal imaging camera (Figure 3c). The camera distance from the sample was approximately 0.6 m. The pictures were taken at full camera resolution of 640 × 320 pixels. The emissivity coefficients of the tested samples were determined by comparing the measurement of the average temperature on the surface of each sample measured by the thermal imaging camera with the actual value of the temperature set in the climatic chamber.

### 2.5. Characterization Surface Morphology

#### 2.5.1. Scanning Electron Microscopy

Graphene oxide deposited on screw was investigated by scanning electron microscopy (SEM) using a Quanta 250 FEG SEM, FEI, Hillsboro, OR, USA. SEM images were acquired using a backscattered detector (ETD-BSE, FEI, Hillsboro, OR, USA) with an accelerating voltage of 2–5 kV for GO and 5–10 kV.

#### 2.5.2. Confocal Microscopy

The measurements of the changes in surface roughness of stainless steel 1.4541 as a result of plasma cleansing and GO deposition were made using a confocal microscope Zeiss LSM 700 (Carl Zeiss Microscopy, Jena, Germany). The experimental parameters were laser wavelength 405 nm, pinhole 0.5 AU (Airy Unit), and gain 416 nm. Five measurements for each sample were made.

#### 2.5.3. Fourier-Transform Infrared Spectroscopy

Measurements of the spectra of GO in the infrared range were made using the FTIR technique in reflection mode. The measurements were made to confirm GO deposition on the stainless steel 1.4541 surface (BM). For FTIR measurements, a Thermo Scientific Nicolet iN10 with a DTGS detector (ThermoFisher SCIENTIFIC, Waltham, MA, USA) was used. Spectra were recorded nine times in different locations with 126 scans in a range of 675–4000 cm^−1^ with a resolution of 4 cm^−1^.

## 3. Results

### 3.1. Surface Morphology

**Figure 4:**
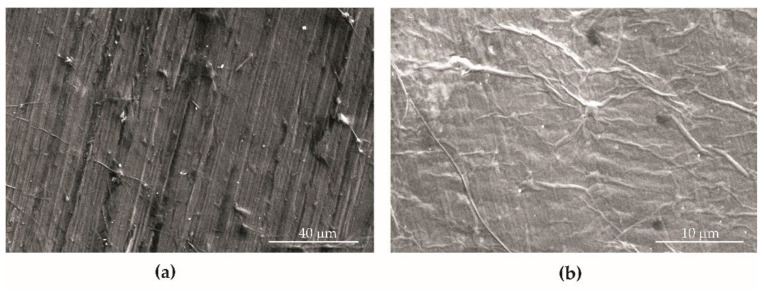
SEM (Scanning Electron Microscope) images of the surface of BM (**a**) and BM + GO (**b**) samples.

The analysis of the surface morphology of BM (Figure 4a) and BM + GO (Figure 4b) samples showed that graphene oxide flakes fill and smooth the structural notches, i.e., cracks formed during the sheet rolling process. As a result, a reduction in surface roughness after graphene oxide deposition of about 21.9% (Ra^1^) and 32.8% (Rz^2^) was observed (Table 2).

**Figure 5:**
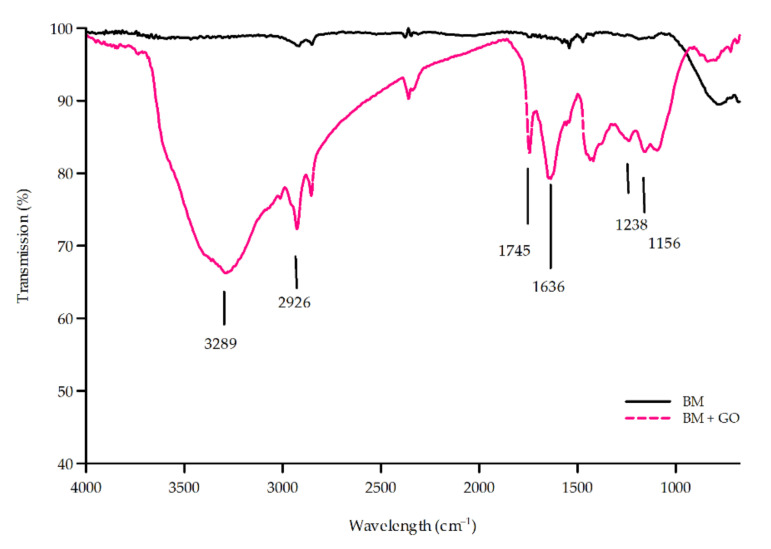
FTIR (Fourier-transform Infrared Spectroscopy) spectra of the BM and BM + GO samples.

In order to confirm the presence of graphene oxide on the surface of austenitic steel 1.4541, FTIR studies were performed which showed a positive result when applying GO on the surface (BM + GO). Figure 5 shows typical absorption bands characteristic for oxidized domains of GO [16].

The GO samples (BM + GO) have absorption peaks at 3289 cm^−1^, 2976 cm^−1^, 1745 cm^−1^, 1636 cm^−1^, 1238 cm^−1^, and 1156 cm^−1^ corresponding to the vibrations of the C–O bond, C=C bond, C=O bond, aliphatic C–H, and O–H bond, respectively. This shows that the surfaces examined have oxygen-containing groups in the BM + GO sample, which proves successful deposition of the GO as compared to the BM sample spectrum.

### 3.2. Static Tensile Tests

Static tensile tests were performed for three temperature levels 20, 100, and 200 °C. The mean results of the five trials along with the standard deviations are listed in Table 3. A decrease (occurring at the limit of statistical error) in the values of tensile strength Rm (1.53–2.56%), yield strength Re (2.97–5.65%), and Young’s modulus E (2.93–8.89%) was observed in all the samples tested in comparison with the untreated ones. The exception was an 8.89% decrease in the elastic modulus at 200 °C of BM + GO samples compared to BM samples.

**Figure 6:**
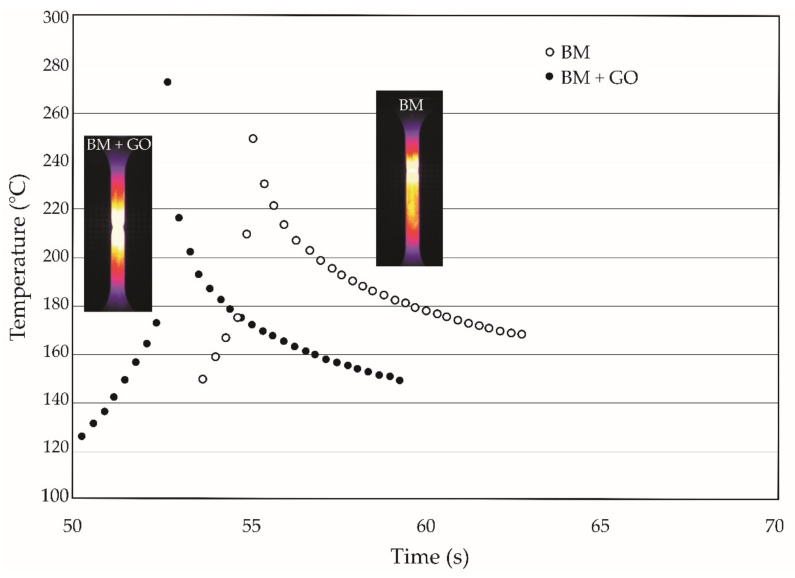
Temperature distribution during static tensile failure test of BM and BM + GO.

The maximum temperature during the static tensile test at the point of breakage was 9.4% lower for BM specimens as compared to BM + GO specimens. It was also observed that after tensile failure, the cooling process of BM + GO samples was faster (Figure 6). The recorded difference is probably due to the blocking of heat propagation by the graphene flake layer.

### 3.3. Fatigue Tensile Tests

Fatigue life tests (at 20, 100 and 200 °C) were performed at σ_max_ levels of 0.9, 0.8, 0.7, and 0.6 Rm (540, 500, 420 and 370 MPa). The material strength limit Rm was determined experimentally and was 600 MPa (Table 3). For elevated temperature tests, the tensile strength limit Rm must be higher than the specified cycle stress. Thus, the fatigue life was not tested at 0.9 for specimens tested at 100 °C and 0.9 and 0.8 for specimens tested at 200 °C.

**Figure 7:**
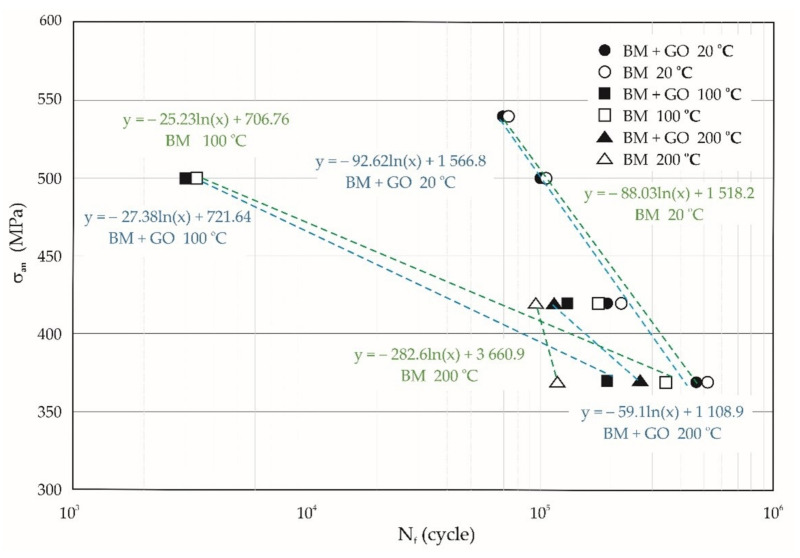
Wöhler plot of the limited range of BM and BM + GO samples at different temperature ranges of 20, 100, and 200 °C.

Averaged results of fatigue life tests (from four measurements) in the temperature range of 20, 100, and 200 °C were used to prepare the Wohler diagram (Figure 7).

No significant effect of graphene oxide deposition on fatigue life was observed at 20 °C. Test results obtained at 20 °C, presented as curves in Figure 7, show a slight (12% (370 MPa), 14% (420 MPa), 2% (500 MPa)) increase in fatigue life of BM specimens in comparison with BM + GO specimens. The difference in fatigue life of BM specimens compared to BM + GO specimens tested at 100 °C was much higher at 77% (370 MPa), 37% (420 MPa), and 8% (500 MPa). However, surprising results on the lifetime of BM + GO samples were obtained at 200 °C. The tests showed that the fatigue life at 200 °C of BM + GO specimens is higher than that of BM specimens by 135% and 21% at nominal stress amplitudes of 370 and 420 MPa, respectively.

**Figure 8:**
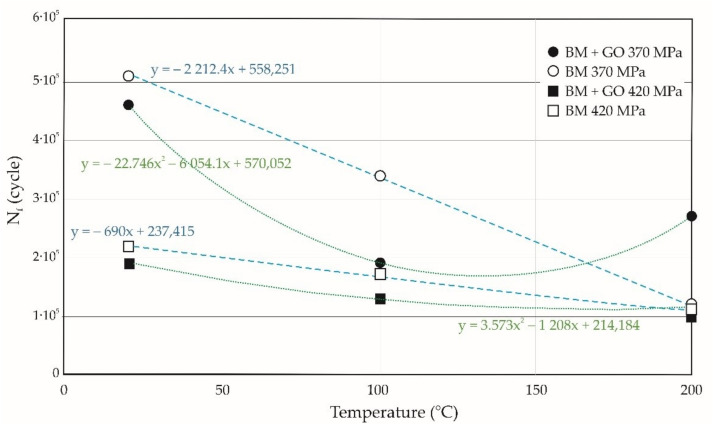
Fatigue life of specimens with and without deposited graphene oxide at 20, 100, and 200 °C performed for maximum stresses of 370 and 420 MPa.

In Figure 8 the curves of fatigue life changes with respect to temperature at 20, 100, and 200 °C performed at a maximum stress level σ_max_ of 370 and 420 MPa are presented. At 20 °C and 100 °C, an increase in the fatigue life of BM specimens compared to BM + GO was observed. However, at 200 °C the opposite situation was observed. There was an increase in the fatigue life of BM + GO samples compared to BM samples. In general, the study showed that as the temperature increases, the fatigue life of BM specimens decreases, in contrast to BM + GO specimens. This may be due to the change in the structural properties of graphene oxide flakes as a result of temperature changes and variable loads.

**Figure 9:**
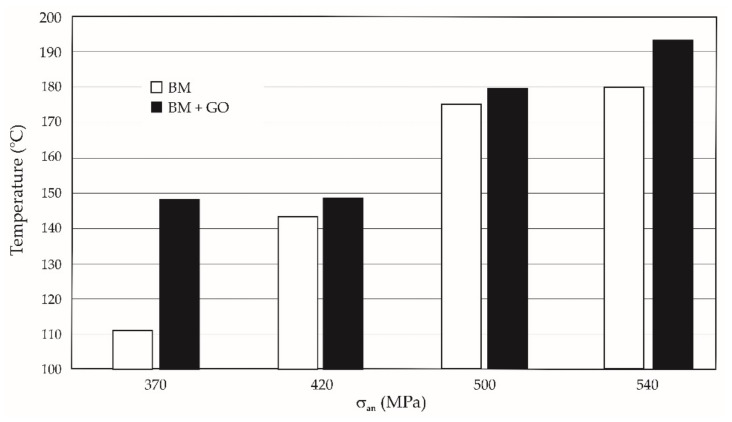
Temperature difference during fatigue rupture of BM and BM + GO samples.

During the fatigue life tests, a higher temperature of 3–26% of the BM + GO specimens was also observed in comparison with BM specimens during final failure. The deposited graphene oxide layer provided an insulating layer, which made the sample heat up more slowly (Figure 9).

#### 3.3.1. Microfractography of Fatigue Fracture Surfaces

**Figure 10:**
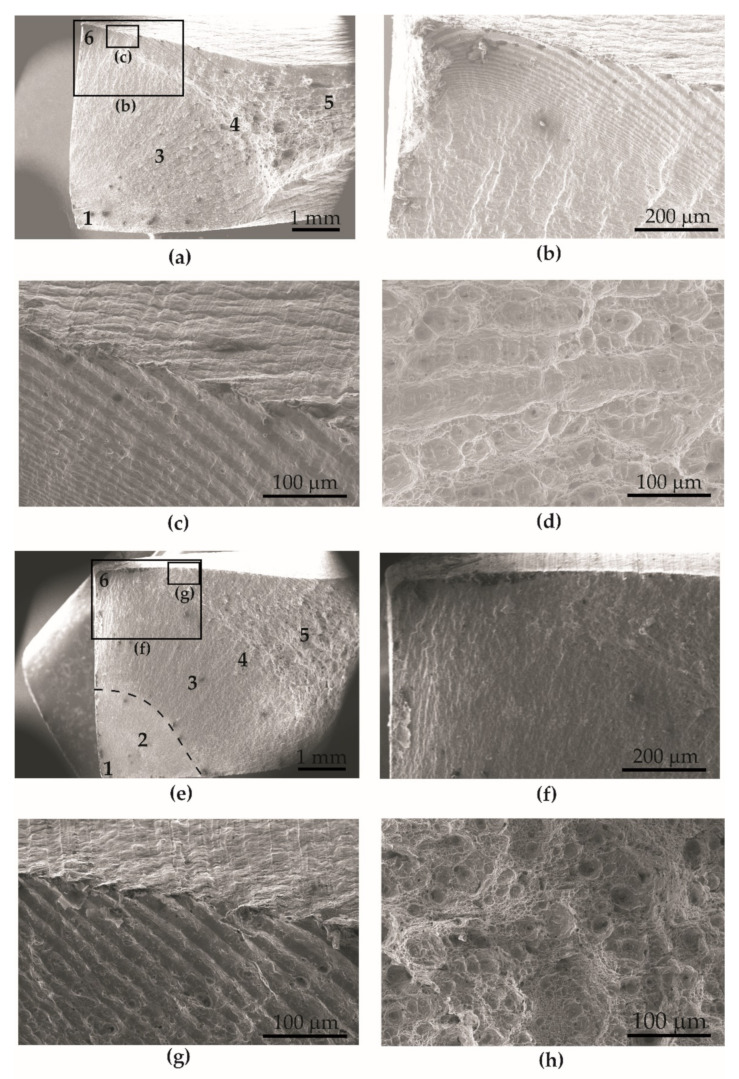
Microfractography of the fatigue fracture of BM (**a**–**d**) and BM + GO (**e**–**h**) samples. Single-focus fatigue crack initiation (1), focal zone (2), fatigue zone (3), transition zone (4), residual zone (5), faults (6).

Microfractography of the surface of fatigue fractures of BM specimens and BM + GO specimens observed by scanning electron microscopy SEM revealed the sites of crack initiation (Figure 10a,e), steady-state crack development (Figure 10b,c,f,g), and pits in the residual zone (Figure 10d,h). Unifocal fatigue crack initiation (1) of BM (Figure 10a–d) and BM + GO (Figure 10e–h) samples occurred similarly—mainly at the edge of the samples (Figure 10a,e).

Microfractography analysis of the fatigue fractures showed a shiny zone (2) around the fracture initiation focus in the BM + GO specimens. The next stage of sample failure was the transition zone (4) separating the fatigue zone (3) from the residual zone (5), where rapid specimen damage occurred [17]. On the opposite side to the crack initiation site, faults appeared (6) (Figure 10b,f). However, they were more pronounced on the lateral edge for BM samples (Figure 10a,b) than for BM + GO samples (Figure 10e,f). This may have been due to the reduction in surface roughness of the samples after graphene oxide deposition (Section 3.1). However, at a distance of about 1 mm from the lateral edge of the fracture, faults were visible for both types of samples, BM (Figure 10c) and BM + GO (Figure 10g).

The final stage of damage occurred as a consequence of the separation characteristic for static cracking (Figure 10d,h). There was an increase in cavities and craters (BM—Figure 10d and BM + GO—Figure 10h). Observation of the sample shows that in the hollow zone the breakthroughs were of a typically ductile character and so-called honeycombs were formed. The craters located in the center of the sample were large and oval, while at the edge of the sample the residual zone was elongated and characterized by a smaller depth.

#### 3.3.2. Microfractography of Graphene Oxide Cracks on the Steel Surface

**Figure 11:**
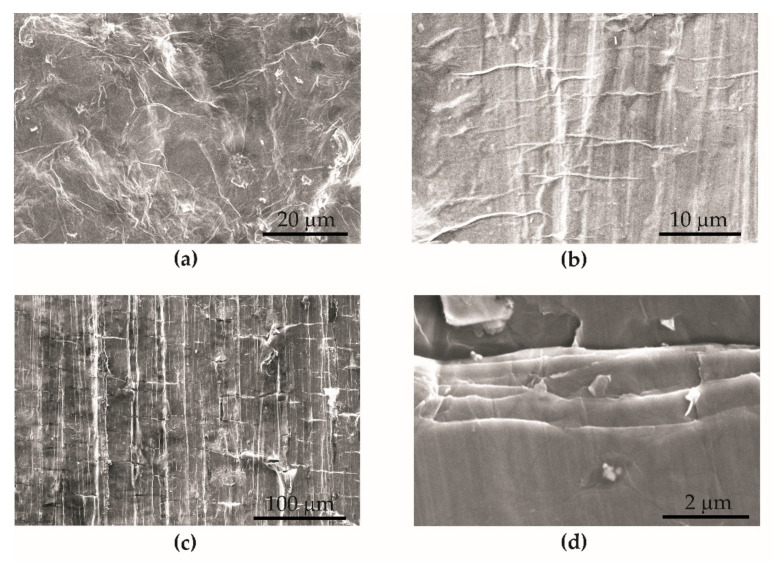
Microfractography of deposited graphene oxide on the surface of steel 1.4541 BM + GO specimens during fatigue tests. Before (**a**) during (**b**,**c**) and after the tests (**d**).

The deposited graphene oxide flakes formed a uniform surface on the BM + GO samples (Figure 11a). In the initial stage of fatigue cycling during the tests, extrusion-shaped lines arranged perpendicular to the force of the fatigue machine actuator and parallel to the fracture front were visualized on the surface of graphene flakes (Figure 11b). Then, as the number of cycles increased, parallel cracks of the deposited graphene oxide layer occurred at the extrusion sites (Figure 11c,d). This probably affects the performance properties (strength, durability, temperature changes) of the BM + GO samples.

## 4. Discussion

The graphene oxide coating applied on flat samples made of 1.4541 steel had a positive effect on surface smoothing. The arithmetic mean of the profile deviation (Rz—maximum peak to valley height of the profile) for BM + GO was 32.8% lower than for BM samples (without GO coating) (Figure 4). It was observed that the deposited GO layer on the specimens was generally durable. However, at the final stage of fatigue life testing, parallel cracking of the coating occurred (Figure 11).

As the test temperatures of 20, 100, and 200 C increased along with effects on the mechanical properties of BM and BM + GO specimens, there was a typical decrease in yield stress, short-term tensile strength, and Young’s modulus. The GO coating at elevated temperature acted as a thermal barrier causing an increase in the temperature of the samples at the break point of 9.4% (Figure 6).

Fatigue tests of the samples for the assumed maximum cycle stress levels confirmed insignificant differences in the durability of BM and BM + GO samples at 20 °C (Figure 7). A decrease in the fatigue life of BM and BM + GO samples occurred at 100 °C; differences reached 77% depending on the stress level of the fatigue cycle. The resulting fatigue damage at the highest maximum cycle stress σ_max_ = 540 MPa led to an 8% reduction in the life difference of BM and BM + GO specimens. On the other hand, the fatigue cracking of the specimens at the lowest fatigue cycle stress σ_max_ = 370 MPa developed in a stable manner, and thus the difference in durability was 77%.

The decrease in fatigue life of the BM + GO samples tested at 20 and 100 °C occurred at all stress levels in the maximum fatigue cycle. However, at 200 °C, the fatigue life of BM + GO specimens was higher than that of BM specimens at maximum cycle stress levels σ_max_ = 370 and 420 MPa. Undoubtedly, this is a surprising result, but it is probably related to the change in properties of the GO layer (at the highest temperature of the tests, 200 °C) and concerns a more favorable system of residual stresses occurring in the GO layer.

After analyzing the test results, it should be noted that the graphene oxide coating alone deposited on the steel did not unequivocally increase its fatigue life. Therefore, it is proposed that an additional strengthening treatment be carried out such as mechanical shot peening, which allows the deposited graphene oxide coating to be introduced into the surface layer and takes advantage of the unique properties of graphene to unequivocally increase durability, as the authors presented in [12].

## 5. Conclusions

The analysis of the results of investigations into the influence of the coating of deposited graphene oxide on the fatigue life of austenitic steel 1.4541 in different temperature ranges showed the following.

(1) Decrease (at the limit of statistical error) of tensile strength Rm by 1.53–2.56%, yield strength Re by 2.97–5.65% and Young’s modulus E by 2.93–8.89% for samples with a deposited graphene oxide layer (BM + GO) in comparison with untreated samples (BM).
(2) Increase in temperature of BM + GO samples during breaking of 9.4% in static tensile tests and 3–26% in fatigue tests.
(3) Decrease in fatigue life of BM + GO specimens compared to BM specimens regarding temperature stress level, at respective levels of
  ∘ 20 °C: 12% for 370 MPa, 14% for 420 MPa, 2% for 500 MPa and
  ∘ 100 °C: 77% for 370 MPa, 37% for 420 MPa, 8% for 500 MPa.
(4) Increase in fatigue life at 200 °C of BM + GO samples by 135% and 21% at nominal stress amplitude of 370 and 420 MPa, respectively.
(5) The formation of faults at the lateral edge for BM specimens, which may be due to higher surface roughness.
(6) Parallel cracking of graphene flakes on the steel surface during fatigue tests.

## 6. Patents

The processing of elements presented in this work consisting of plasma cleaning and surface activation, application of graphene oxide, vacuum drying, and mechanical shot peening was called hybrid graphene treatment and is presented in patent application P. 438715.

## Figures and Tables

**Table 1:** Fatigue test load levels (Rm—Ultimate tensile strength).

Fatigue Test Parameters	Load Levels			
	Rm = 0.9	Rm = 0.8	Rm = 0.7	Rm = 0.6
Maximum stress (MPa)	540.1	500.0	420.0	370.0
Maximum force (kN)	33.66	31.16	26.18	23.06
Minimum force (kN)	3.37	3.12	2.62	2.31
Average force (kN)	18.51	17.14	14.40	12.68
Amplitude of cycle load (kN)	15.15	14.02	11.78	10.38

**Table 2:** Surface roughness of BM and BM + GO (Base Material + Graphene Oxide) samples (mean of 5 measurements) with standard deviation.

Surface Type	Mean Value Ra ^1^ (µm)	Mean Value Rz ^2^ (µm)
BM	1.12 ± 0.27	6.15 ± 1.59
BM + GO	0.895 ± 0.16	4.13 ± 0.35

^1^ Ra—arithmetic average. ^2^ Rz—maximum peak to valley height of the profile.

**Table 3:** Static tensile test results.

Sample Type	Temperature (°C)	Tensile Strength Limit Rm (MPa)	Young’s Modulus E (MPa)	Yield Strength Re (MPa)
BM	20	600.1 ± 1.5	196,156.9 ± 4697	350.7 ± 2.5
	100	527.5 ± 10.2	194,624.6 ± 8359	325.7 ± 14.3
	200	464.5 ± 0.4	194,332.8 ± 8065	292.0 ± 1.0
BM + GO	20	590.9 ± 1.9	190,415.2 ± 5253	340.3 ± 2.5
	100	514.0 ± 3.0	188,717.4 ± 1259	307.3 ± 2.1
	200	453.2 ± 1.7	177,055.6 ± 1164	282.0 ± 2.6

## Data Availability

Data are contained within the article.
